# Host-resistance in *Allium* genotypes against pantaphos producing *Pantoea ananatis*

**DOI:** 10.3389/fpls.2025.1628122

**Published:** 2026-02-10

**Authors:** Brendon K. Myers, Navjot Kaur, Michelle P. MacLellan, Ronald Gitaitis, Angelo Antonio Manzatto, Adriano Ferrasa, Elkin Fernando Rodas-Mendoza, Jhon Jairo Giraldo-González, Roberto H. Herai, Bhabesh Dutta

**Affiliations:** 1 Department of Plant Pathology, University of Georgia, Tifton, GA, United States; 2 Bioinformatics and Computational Biology Laboratory (LBBC), Postgraduate Program in Applied Computing (PPGCA), Department of Informatics (DEINFO), Universidade Estadual de Ponta Grossa (UEPG), Ponta Grossa, Paraná, Brazil; 3 Research Group in Phytochemistry and Molecular Biology (FITOBIOMOL), Faculty of Basic Sciences, Francisco de Paula Santander University, Cúcuta, Colombia; 4 Laboratory of Bioinformatics and Neurogenetics (LaBiN), Graduate Program in Health Sciences (PPGCS), Pontifícia Universidade Católica do Paraná (PUCPR), Curitiba, Paraná, Brazil; 5 Experimental Multiuser Laboratory (LEM), Graduate Program in Health Sciences (PPGCS), Pontifícia Universidade Católica do Paraná (PUCPR), Curitiba, Paraná, Brazil

**Keywords:** *Allium*, *Pantoea ananatis*, onion center rot, resistance, plant disease, *in vivo* transcriptome

## Abstract

**Introduction:**

Onion (*Allium cepa* L.) is a globally important crop severely affected by *Pantoea ananatis*, the causal agent of onion center rot (OCR). The pathogen’s virulence is driven by the chromosomally located HiVir cluster, which produces the phytotoxin pantaphos. Despite its economic significance, resistant *Allium* genotypes against *P. ananatis* have not been identified.

**Methods:**

We screened 982 *Allium* genotypes under field conditions to evaluate resistance against pantaphos-producing *P. ananatis* and conducted *in vivo* transcriptome sequencing of resistant vs. susceptible genotypes under controlled growth-chamber conditions.

**Results:**

Only one genotype, DPLD 19-39, exhibited consistent resistant phenotype, displaying reduced foliar necrosis and bulb rot. Transcriptomic analyses revealed differential regulation of key defense-associated pathways, including cell wall reinforcement, oxidative stress regulation, and programmed cell death.

**Discussion:**

These findings provide the first evidence of a resistant *A. cepa* genotype against pantaphos-producing *P. ananatis*. The identified molecular responses highlight potential targets for developing onion cultivars with durable resistance to onion center rot.

## Introduction

Onion (*Allium cepa* L.) belongs to the family Amaryllidaceae and is a biennial plant primarily cultivated annually for its edible bulb (Rabinowitch and Brewster, 1989). *Allium cepa* genotypes are highly susceptible to *Pantoea ananatis* (PA), a bacterium that causes onion center rot (OCR). Severe infections of OCR in major onion-producing regions, like the Vidalia onion region in Georgia, have led to significant economic losses, sometimes amounting to millions of dollars in revenue (Gitaitis and Gay, 1997; Schwartz and Mohan, 2008; Coutinho and Venter, 2009; Dutta, 2020). In addition to other members of the *Allium* genus, PA infects a wide range of economically important crops globally. It was first reported as fruitlet rot in pineapple (Philippines) (Serrano, 1928), and since then, it has been identified as an epiphyte or endophyte on both dicots and monocots in both the United States and beyond on a wide-range of crops including honeydew melon, cantaloupe, onion, Sudan grass, eucalyptus, rice, netted melon, maize, and sorghum (Bruton et al., 1991; Coutinho and Venter, 2009; Kido et al., 2008; Alippi and López, 2010; Cota et al., 2010). In the United States, PA has been reported from various onion-growing regions (Georgia, Colorado, Michigan, New York, and Pennsylvania) as causal agents of OCR (Wells et al., 1987; Gitaitis and Gay, 1997), PA can be seed-borne and seedling-transmitted, but *Thrips tabaci*-mediated transmission is more common and epidemiologically significant, particularly in regions like the southeastern United States (Gitaitis et al., 2002; Dutta et al., 2014a). These thrips species can acquire epiphytic PA populations from various environmental host plants and transmit the pathogen to healthy onion seedlings. PA can also invade onion foliage through wounds, leading to water-soaked lesions, blighting, and wilting of the infected leaves. Foliar colonization and infection can result in bacterial movement to onion bulbs that may result in bulb infection (Schwartz and Mohan, 2008; Stice et al., 2018). The virulence of PA is attributed to the chromosomally localized “HiVir” gene cluster, which encodes the phosphonate phytotoxin pantaphos (Asselin et al., 2018; Polidore et al., 2021). Pantaphos disrupts metabolic processes in the plant, resulting in cell death and necrosis (Asselin et al., 2018; Polidore et al., 2021). Cell death in *Allium* tissues leads to a significant challenge; however, as the tissues are rich in thiosulfinate compounds, which serve as a natural antimicrobial; the plasmid-borne thiosulfinate tolerance (*alt*) gene cluster allows PA to thrive and proliferate in disrupted *Allium* tissues by reducing toxic thiol stress (Stice et al., 2020; Stice et al., 2021). Despite the discovery of these virulence factors, there has been little progress in determining host-resistance against pantaphos-producing PA in Allium genotypes. In this study, we conducted a comprehensive screen of various *Allium* genotypes for resistance to PA. In addition, through transcriptome analysis of a PA-susceptible (Sweet Harvest) and a resistant genotype (DPLD 19-39), we identified differentially expressed transcripts potentially involved in pathogen resistance mechanisms against PA. However, it remains unclear how these specific genotypes of *Allium* resist PA infection at the molecular level. Moreover, although genes related to cell-wall remodeling and reactive oxygen species (ROS) are broadly conserved in *Allium* species, it is yet to be clear as to if the *Allium* genotypes reacts differentially to the pantaphos toxin specifically, or if the mechanism of resistance is through a more generalized strategy against the bacterium itself. In this study, we aim to determine the molecular mechanisms underlying the resistance observed in genotype DPLD 19–39 against PA, with particular focus on defense pathways activated during infection.

## Materials and methods

### Bacterial strain, identification, culturing

A PA strain, PNA 97–1 was used in this study that was isolated from *A. cepa* in 1997 and is a well-characterized pantaphos-producing aggressive strain (Gitaitis and Gay, 1997). Inoculum was prepared by transferring single colonies from 24 h-old cultures on nutrient agar (NA) medium to nutrient broth (NB). The broth was shaken overnight on a rotary shaker (Thermo Scientific, Gainesville, FL) at 180 rpm. After 12 h of incubation, 1 ml of each bacterial suspension was centrifuged at 5,000 × g (Eppendorf, Westbury, NY) for 2 min. The supernatant was discarded, and the pellet was re-suspended in 0.1 M phosphate buffer saline (PBS). Inoculum concentration was adjusted using a spectrophotometer (Eppendorf, Westbury, NY) to an optical density of 0.3 at 600 nm [≈1 × 10^8^ colony forming units (CFU)/ml].

### Phenotypic assessment of *PA* PNA 97–1 on *Allium* genotypes

Foliar pathogenicity and aggressiveness of PA 97–1 were determined under field and controlled greenhouse conditions. Artificially infested onion seeds of *Allium* genotypes (n=982) were used in the field experiment. This was done to ensure maximum exposure of the pathogen to the *Allium* genotypes, starting from the seed and seedling stages. Infested seeds were generated separately for each *Allium* genotype by exposing them to inoculum (at a concentration stated above) via vacuum infiltration as per the manufacturer’s instruction for 1 min. An additional cycle of vacuum infiltration for 1 min was also conducted. Ten seeds in three replicates for each *Allium* genotype were planted in a row at a 10-cm spacing. These seeds were allowed to germinate and grow to at least the four true-leaf stage. The tallest leaf of each *Allium* genotype was inoculated using a cut-tip method as described previously (Dutta et al., 2014a). Briefly, a wound was created by cutting the central leaf (2 cm from the apex) with a sterile pair of scissors. A 10 µl drop of a bacterial suspension containing 1×10^8^ CFU/ml was placed at the cut end. One plant at each end of the row was inoculated with sterile water as negative controls for foliar inoculation for each replicate or plot/*Allium* genotype. The rest of the plants were inoculated as described above. A susceptible *Allium* genotype, Sweet Harvest, was used in this experiment. The field was left without management against weeds and thrips to further pathogen spread and disease development. Plants in the field were assessed for foliar symptoms at least 4 times (1-day post-inoculation, 1-week post-inoculation, 19-days post-inoculation, and 30-days post-inoculation).

Based on the initial field screen four *Allium* genotypes, DPLD-19-39, Sweet Harvest, Zhang Qiu Da Cong, and Koshizu Nebuka, were evaluated for foliar disease severity under greenhouse conditions. For the greenhouse studies, seedlings for each *Allium* genotype were established in plastic pots (T.O plastics, Clearwater, MN) with dimensions of 9 cm × 9 cm × 9 cm (length × width × height) containing a commercial potting mix (Sta-Green, Rome, GA). The seedlings were maintained at 25-28 °C and 70-90% relative humidity with a light:dark cycle of 12h:12h. Bacterial strain (PNA 97-1) was maintained on NA plates, and inoculum was generated as described above. Once the primary leaf of each *Allium* genotype reached 9 cm, seedlings were inoculated using a cut-tip method as described previously (Dutta et al., 2014a). Seedlings were inoculated with sterile water using the same methodology as above served as controls. A susceptible *Allium* genotype, Sweet Harvest, was also used in this experiment. Ten replicates per genotype was used in a single experiment, and two independent experiments (GH-1 and GH-2) were conducted with selected genotypes.

Based on the greenhouse experiments, one *Allium* genotype (DPLD 19-39) was selected for growth chamber assessment and was compared with a susceptible check (Sweet Harvest). Seedlings for these two genotypes were inoculated at a 4-true-leaf growth stage using a protocol described above. Disease assessments were done according to the protocol stated below.

The pathogenicity and aggressiveness of PA strain PNA 97–1 were determined based on the lesion length on each *Allium* genotype, measured with a ruler at different assessment periods. The lesion length was recorded weekly for three weeks after foliar inoculation for the field experiment. The lesion size was analyzed using the rating scale for the field evaluations for *Allium fistulosum*, where lesion size was categorized from 0 (no lesion) to 6 (>20.1 cm or dead), and for *Allium cepa*, where the lesion size was categorized from 0 (no lesion) to 10 (>40 cm or dead).

For greenhouse and growth chamber evaluations, the percentage lesion length relative to the average length of the leaf for that genotype was calculated at 12 days post-inoculation. The area under the lesion progress curve (AULPC) was calculated for each genotype and compared between each other and the controls. Analysis of variance (ANOVA) was determined for percent lesion length in R (R version 4.3.0), and Tukey’s honestly significant difference (HSD) test was used to determine the mean separation for different genotypes.

### Phenotypic assessment of bulb infection on selected *Allium* genotypes against PA PNA 97–1 invasion

Bulbs of DPLD 19–39 and Sweet Harvest were harvested after three months of growth under controlled conditions in a growth chamber. The seedlings were maintained at 25-28°C and 70-90% relative humidity with a light:dark cycle of 12h:12h. The outer tunic layer was carefully removed, surface-sterilized by spraying 70% ethanol followeed by air-drying for 15 mins. After air-drying, scales were carefully removed, and using a sterile inoculation loop, bacterial ooze was scooped, ten-fold serially diluted, and spread-plated onto a semi-selective medium, PA-20 (Goszczynska et al., 2007). After a period of incubation (7 days), small colonies were enumerated. Representative colonies were also assayed with PA HiVir-specific PCR assay (Shin et al., 2025).

In addition to the evaluation of inter-scale bacterial colonization, remaining healthy appearing bulbs from both genotypes were surface sterilized with 70% ethanol after the removal of tunic layers. Each bulb was placed on a plate containing two layers of paper towel pre-moistened with sterile water. Onion bulbs were inoculated longitudinally at the shoulder with a syringe and a sterile needle containing a volume of 400 µl (1×10^8^ CFU/ml) (Schroeder et al., 2010). Special attention was given to the uniformity of depth of inoculation into each bulb, which was ascertained by placing a thin rubber stopper in the needle. Following inoculation, bulbs were incubated at 25 °C in an aluminum tray. After a week of incubation, bulbs were sliced vertically alongside the inoculation site, and the weight of the whole bulb and symptomatic scales with necrotic lesions (and visual rot) were measured and recorded.

### Transcriptome analysis of DPLD 19–39 vs Sweet Harvest

Host plants were grown under greenhouse conditions, as previously described. Plants were inoculated with PA strain PNA 97–1 using two treatments for each genotype: bacterial suspension (PA-positive) and PBS as a negative control. After 24 hours, foliar tips were excised approximately 1 cm below visible lesions and immediately flash-frozen in liquid nitrogen for RNA extraction. Total RNA was extracted using the RNeasy Plant Mini Kit (Qiagen, Germantown, MD, USA) according to the manufacturer’s instructions. RNA sequencing was performed by Azenta Life Sciences (South Plainfield, NJ, USA). Library preparation utilized the NEBNext Ultra II RNA Library Prep Kit for Illumina with Poly(A) selection, and sequencing was conducted on an Illumina platform (Illumina Inc., San Diego, CA, USA) to produce 2×150 bp paired-end reads, generating approximately 350 million reads across all libraries.

### Differential gene expression analysis

The RNA-seq data was analyzed following a standard bioinformatics pipeline to perform quality control of raw sequencing reads using FastQC (Andrews, 2010), which assesses sequencing quality metrics related to per-base quality scores, GC content, sequence contaminants, and adapter presence. The low-quality reads were then removed using Cutadapt (Martin, 2011). Next, reads were aligned against the *A. cepa* genome and corresponding gene models (Finkers et al., 2021) using the STAR aligner (Dobin et al., 2013). Mapped reads contained in binary bam files were then processed using featureCounts software (Liao et al., 2014) to create a file representing a matrix of gene expression levels.

The matrix of absolute read counts for each gene was then subjected to a normalized gene expression analysis with statistical significance using the Bioconductor (Gentleman et al., 2004) package DESeq2 (Love et al., 2014). Within this process, the biological replicates for each sample were explicitly assigned to their respective experimental conditions (e.g., DPLD 19–39 control, Sweet Harvest inoculated) to establish the design for statistical comparisons. The DESeq2 statistical model then leverages the variance across the full set of replicates within each group to accurately estimate gene-wise dispersion and test for significant differences in expression. In this step, read counts data were transformed into a DESeq2 dataset for differential expression analysis, including pre-filtering low-count genes to identify significant results based on adjusted p-values (Benjamini-Hochberg statistical method). Genes with adjusted p-values of less than 0.05 were considered significant in this study. This threshold indicates less than a 5% chance that the observed results were due to random variation alone.

### Gene ontology and pathway analysis

Gene ontology annotation and pathway analysis were performed using differentially expressed genes with statistical significance data. Differentially expressed genes were subjected to the software clusterProfiler (Xu et al., 2024). Briefly, clusterProfiler internally utilizes a biological knowledge database, including Gene Ontology and Kyoto Encyclopedia of Genes and Genomes (KEGG), by performing over-representation and gene set enrichment analyses. This analysis facilitated the investigation of associations between specific gene lists or sets of genes and their corresponding biological functions, pathways, and classifications.

## Results

### Field evaluations confirmed that resistance to *PA* PNA 97–1 is rare in *Allium* genotypes

A panel of 982 *Allium* genotypes was screened against PA PNA 97-1, an aggressive pantaphos-containing strain isolated in Georgia (USA) from symptomatic onion. Considerable variations in disease severity were observed across *A. cepa*, *cepa var* or *cepa* subsp. consistency, and *A. fistulosum*. For the *A. cepa*, most genotypes were classified as susceptible, accounting for 92.5% of the total screened genotypes. While some genotypes, such as New Mexico Yellow Grano, Linea 139, and Portuguesa Tardia displayed considerably high foliar disease severity and corresponding higher AULPC values, a smaller proportion (3.2%) displayed significantly lower disease severity and as well as lower AULPC values, including the following genotypes: DPLD 19-39, California Red, 1607 Super Sleeper F1, Red Bermuda, Glory, A5718, and Saturn.

Additionally, thirty-one *A. fistulosum* genotypes were screened in two sets (Set 1: N = 10 genotypes with 5 replicates per genotype; Set 2: N = 21 genotypes with seven replicates per genotype) (**Supplementary Table S1**). The phenotypic screen revealed significant variation in disease severity across *A. fistulosum* genotypes, with the highest disease severity and AULPC were observed in Japanese Bunching Hikari and Hardy Long White (**Supplementary Table S2** and **Supplementary Table S3**). Genotypes such as Feast, Kannon Hosonegi, Winter Snow Foot, and Aigarshu displayed considerably low disease severity and AULPC. Genotypes like Shounai Nebuka Negi, Yakko, Big Buncher, YatabeYaty, and Koshizu Nebuka displayed significantly lower disease severity and AULPC than other genotypes.

In *A. cepa*, most genotypes displayed moderate to high levels of disease severity. In Set 1 (**Supplementary Table S4**), which included ten genotypes with six replicates each, the highest AULPC values were observed for Sweet Spanish Los Animas Special, Yellow Ebenezer, and No. 8656 compared with Yellow Grano, which had significantly lower AULPC values. Similarly, in Set 2 (**Supplementary Tables S5**), eleven genotypes were assessed, with three replicates each. No significant differences in AULPC values were observed among the screened genotypes (2935B, White Portugal, Stuttgarter, Yellow Sweet Spanish Utah Strain, 607 Ebenezer, Calred, Early Crystal 281, Giolla di Rovato da Scattaceto, Early Crystal, White Sweet Spanish, and White Lisbon).

### Greenhouse screening of *Allium* genotypes against PA PNA 97-1

In two independent greenhouse experiments (GH-1 and GH-2), four *Allium* genotypes, DPLD-19-39, Sweet Harvest, Zhang Qiu Da Cong, and Koshizu Nebuka, were evaluated for foliar disease severity. Since there was a significant difference between the two experiments, both experiments were analyzed separately (**Table 1**). The main effects, genotypes (P<0.001), treatment (P<0.001), and the interactions (genotype x treatment), were significant (P<0.001) (**Table 2**). In greenhouse experiment 1 (GH-1), significant effects were observed for genotype (P<0.001), treatment (P<0.001), and their interactions (P<0.001) (**Table 2**). The comparison of foliar disease severity among four *Allium* genotypes (**Table 3**) showed that the genotype Sweet Harvest exhibited the highest mean AULPC value (60.5) compared to DPLD-19-39, Koshizu Nebuka and Zhang Qiu Da Cong. Regarding treatment effects, inoculated plants showed significantly higher AULPC values than the control plants (AULPC; inoculated=61.9 cm vs. control=17.2 cm). The significant genotype x treatment interactions (P<0.01) underscore the differential responses of the genotypes to inoculation with PA strain PNA 97-1. Specifically, Sweet Harvest had the highest mean AULPC value when inoculated (105.5), while Zhang Qiu Da Cong had the lowest (29.7). DPLD-19-39 (62.2) and Koshizu Nebuka (50.1) exhibited intermediate values but were not significantly different from each other.

**Table 1:** Combined ANOVA for two greenhouse experiments.

Factor	*P-*value
Genotype (Var)	<0.001
Treatment (Trt)	<0.001
Experiment (Exp)	<0.001
Var × Trt	<0.01
Var × Exp	0.094
Trt × Exp	0.910
Var × Trt × Exp	0.305

The summary of the analysis of variance (ANOVA) results from two greenhouse experiments evaluating foliar lesion development following inoculation with Pantoea ananatis (PNA 97-1). Significant effects were observed for genotype, treatment, and experimental replicate (P<0.001). The interaction between genotype and treatment (Var × Trt) was also significant (P<0.01), suggesting differential genotype responses to inoculation. However, interactions involving the experimental replicate (e.g., Var × Exp, Trt × Exp) were not statistically significant.

**Table 2:** Analysis of variance (ANOVA) and associated P-values for individual greenhouse experiments (GH-1 and GH-2).

Factor	Greenhouse Experiments (GH)	
	GH-1	GH-2
Genotype (Var)	<0.001	<0.01
Treatment (Trt)	<0.001	<0.01
Var × Trt	<0.001	<0.01

The summary of the analysis of variance (ANOVA) results from two greenhouse experiments evaluating foliar lesion development following inoculation with Pantoea ananatis (PNA 97-1). Significant effects were observed for genotype, treatment, and experimental replicate (P<0.001). The interaction between genotype and treatment (Var × Trt) was also significant (P<0.01), suggesting differential genotype responses to inoculation. However, interactions involving the experimental replicate (e.g., Var × Exp, Trt × Exp) were not statistically significant.

**Table 3:** Comparison of the foliar severity for four *Allium* genotypes under greenhouse conditions.

Factor	Greenhouse experiments (GH)	
	AULPC GH-1	AULPC GH-2
Genotypes (Var)		
DPLD-19-39	45.9 ab	68.2 ab
Sweet Harvest	60.5 a	130.7 a
Koshizu Nebuka	29.2 b	36.2 b
Zhang Qiu Da Cong	22.6 b	93.6 ab
Treatments (trt)		
Control (PBS)	17.2 b	56.3 b
Inoculated (Inoc)	61.9 a	105.0 a
Var × Trt	<0.01	<0.01

A detailed comparison of area under the lesion progress curve (AULPC) values for four *Allium* cepa genotypes: DPLD 19-39, Sweet Harvest, Koshizu Nebuka, and Zhang Qiu Da Cong under inoculated and control treatments in two greenhouse experiments. Sweet Harvest exhibited the highest lesion severity in both GH-1 and GH-2, while DPLD 19-39 and Zhang Qiu Da Cong showed significantly reduced symptoms. Notably, DPLD 19-39 demonstrated lower AULPC values for both control and inoculated treatments compared to Sweet Harvest. The numbers in the table are the mean AULPC values followed by the same letters are not significantly different according to Tukey’s honest significant difference (*P* <0.05).

**Figure 1:**
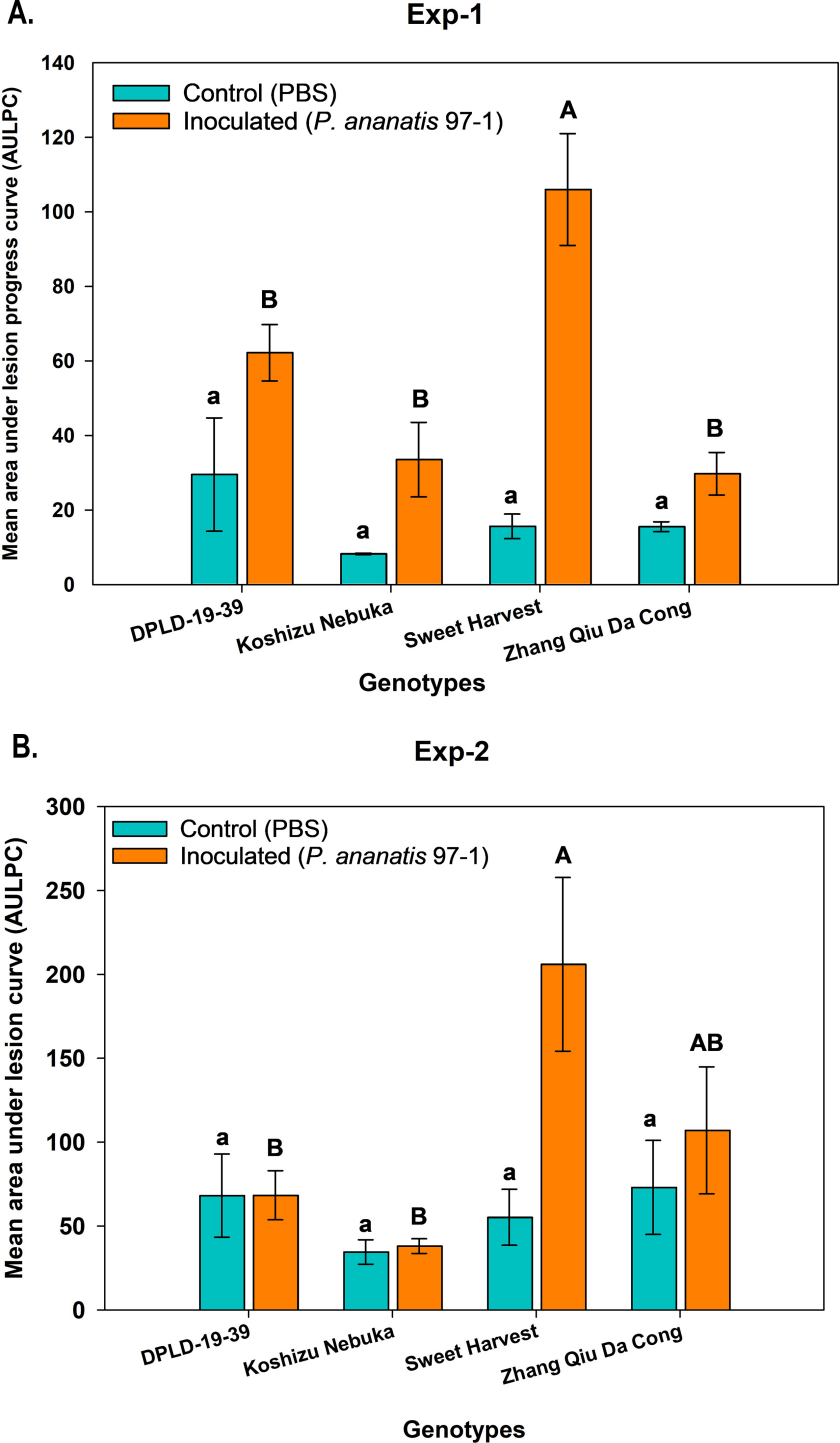
Foliar lesion progression in *Allium cepa* and *Allium fistulosum* genotypes inoculated with *Pantoea ananatis* (PNA 97-1) under greenhouse conditions. Four genotypes were tested: *A. cepa* (DPLD 19-39 and Sweet Harvest) and *A. fistulosum* (Koshizu Nebuka and Zhang Qiu Da Cong). Plants were inoculated with 10 µL of a bacterial suspension (10^8^ CFU/mL) and with phosphate-buffered saline solution -treated plants served as controls. After a 7-day incubation period, the area under the lesion progress curve (AULPC) was calculated from lesion measurements. **(A, B)** AULPC for experiments 1 and 2, respectively. Means on the bars followed by the same letters either upper or lower case are not significantly different according to Tukey’s honest significant difference (*P* <0.05).

In greenhouse experiment 2 (GH-2), similar trends were observed, with significant effects for genotype (P<0.01), treatment (P<0.01), and their interactions (P<0.01) (**Table 2**). Again, Sweet Harvest had the highest mean AULPC value (130.7), compared with DPLD-19-39 (68.2), Koshizu Nebuka (36.2), and Zhang Qiu Da Cong (93.6). In terms of treatment effects, inoculated plants again showed significantly higher AULPC values than controls (AULPC; inoculated = 104.9 vs. control = 56.3) (**Figure 1**).

**Figure 2:**
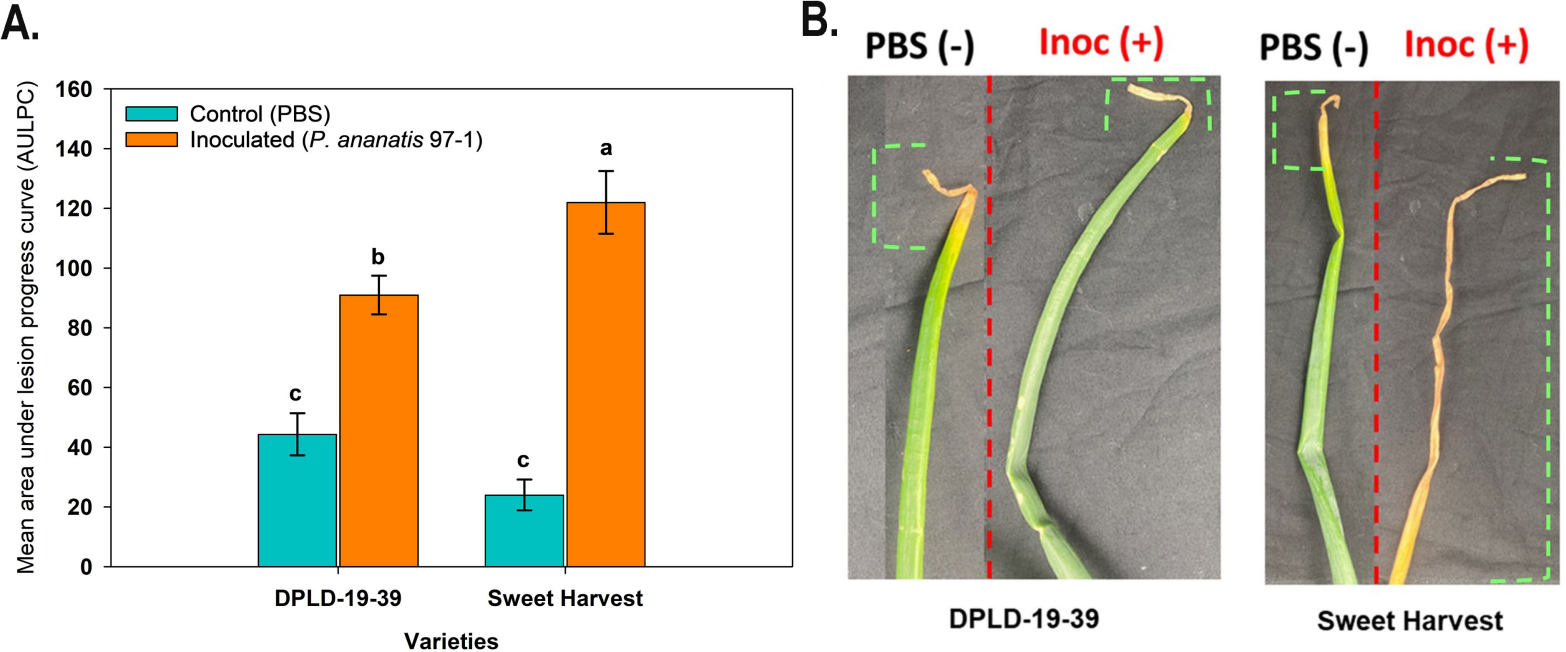
Area under lesion progression curve (AULPC) for DPLD 19-39 and Sweet Harvest in a growth chamber experiment. Onion seedlings were inoculated with *Pantoea ananatis* (PNA 97-1) with a bacterial suspension of 10μl containing 10^8^ colony-forming units/ml and incubated for 14 days. Seedlings inoculated with phosphate-buffered saline (PBS) solution served as negative controls. **(A)** represents the data for lesion length progression over time (every other day for 12 time points post inoculation), and the corresponding AULPC. Twenty replicates per genotype were used in this experiment. The bars followed by the same letters are not significantly different according to Tukey’s "honestly" significant difference (*P* < 0.05) test. **(B)** indicates the foliar lesion in both the negative control (PBS) and inoculated (Inoc (+)) treatments in DPLD 19-39 and Sweet Harvest, respectively. The green brackets in the image indicate the degree or severity of necrotic lesions observed during the experiment.

In a controlled growth chamber experiment, we evaluated the foliar disease severity of *A. cepa* genotypes, DPLD 19-39, and Sweet Harvest against PA PNA 97-1. Growth chamber evaluation further supports to support higher disease severity in Sweet Harvest compared to DPLD 19–39 following PA PNA 97–1 inoculation. The AULPC was determined by measuring the lesion length over a period of two weeks, with linear lesion progression recorded every other day. The AULPC values were significantly higher for PA-inoculated-inoculated Sweet Harvest than for DPLD 19-39. Although inoculation with PBS also resulted in some necrosis in seedlings of both genotypes, AULPC values were significantly lower than the AULPC observed for the inoculated seedlings (**Figure 2**).

### Evaluation of bulb rot symptoms in DPLD 19–39 confirms reduced severity against PA PNA 97-1

**Figure 3:**
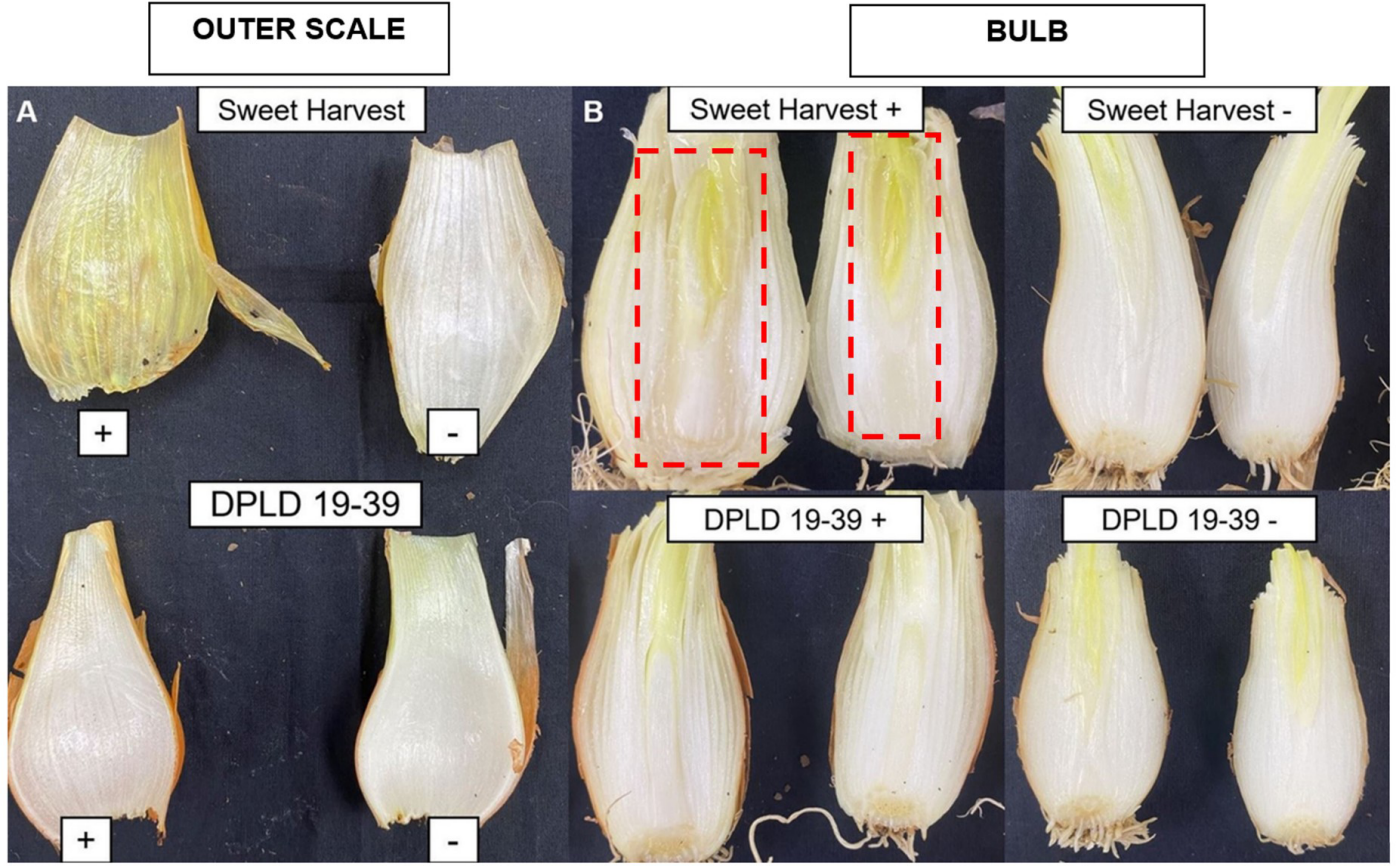
Comparison of onion bulb symptoms in *Allium cepa* genotypes “Sweet Harvest” and “DPLD 19-39” following inoculation with *Pantoea ananatis* (PNA 97-1). **(A)** Outer scale images of two *Allium cepa* genotypes, “Sweet Harvest” and “DPLD 19-39”, taken after 3-months under growth-chamber conditions. Outer scales were removed and assessed for water-soaked bacterial lesion or associated slime. Greasy greenish yellow indicates water-soaked bacterial slime associated lesion. **(B)** Internal bulb rot symptoms in response to PA inoculation to *Allium cepa* genotypes “Sweet Harvest” and “DPLD 19-39. After a week of incubation, bulbs were sliced vertically alongside the inoculation site and the weight of the whole bulb and symptomatic scales with necrotic lesions (and visual rot) was measured and recorded. The signs “+” and “-” denote PA-inoculated and PBS-inoculated onion tissues, respectively. Red dotted lines indicate water-soaked lesions.

Further, we analyzed the ability of *PA* 97–1 to penetrate the onion bulb for both DPLD 19–39 and Sweet Harvest. First, we examined the outer scale of each onion bulb once the foliar lesion experiments were concluded. Sweet Harvest inoculated leaves consistently led to rotting of the attached scales of the associated bulbs.. In contrast, their negative controls were consistently asymptomatic (**Figure 3A**). PA was isolated from the inner scales in 100% of the replicates/samples assayed, which were later confirmed using PA-HiVir specific PCR assay as mentioned above. Isolations made from the inner scales of asymptomatic DPLD 19–39 resulted in bacterial recovery but were not PA, as confirmed by the above PCR assay. To assess if DPLD 19–39 or Sweet Harvest would develop further bulb-rot symptoms, we inoculated PNA 97–1 directly into the bulb and observed that Sweet Harvest consistently developed internal rot symptoms (**Figure 3B**). The Sweet Harvest negative control and DPLD 19–39 treatments did not result in internal bulb rot (**Figure 3B**).

### Transcriptome analysis of DPLD 19–39 vs. sweet harvest genotypes

To investigate whether the resistant DPLD 19–39 and susceptible Sweet Harvest genotypes present different transcriptional responses during infection with PA, we conducted a comprehensive transcriptomic analysis using RNA-seq. Each genotype was sampled under control and PA-inoculated conditions with three biological replicates per treatment. RNA-seq generated an average of ~20 million paired-end reads per sample, with mapping rates of ~90% to the *A. cepa* reference genome.

**Figure 4:**
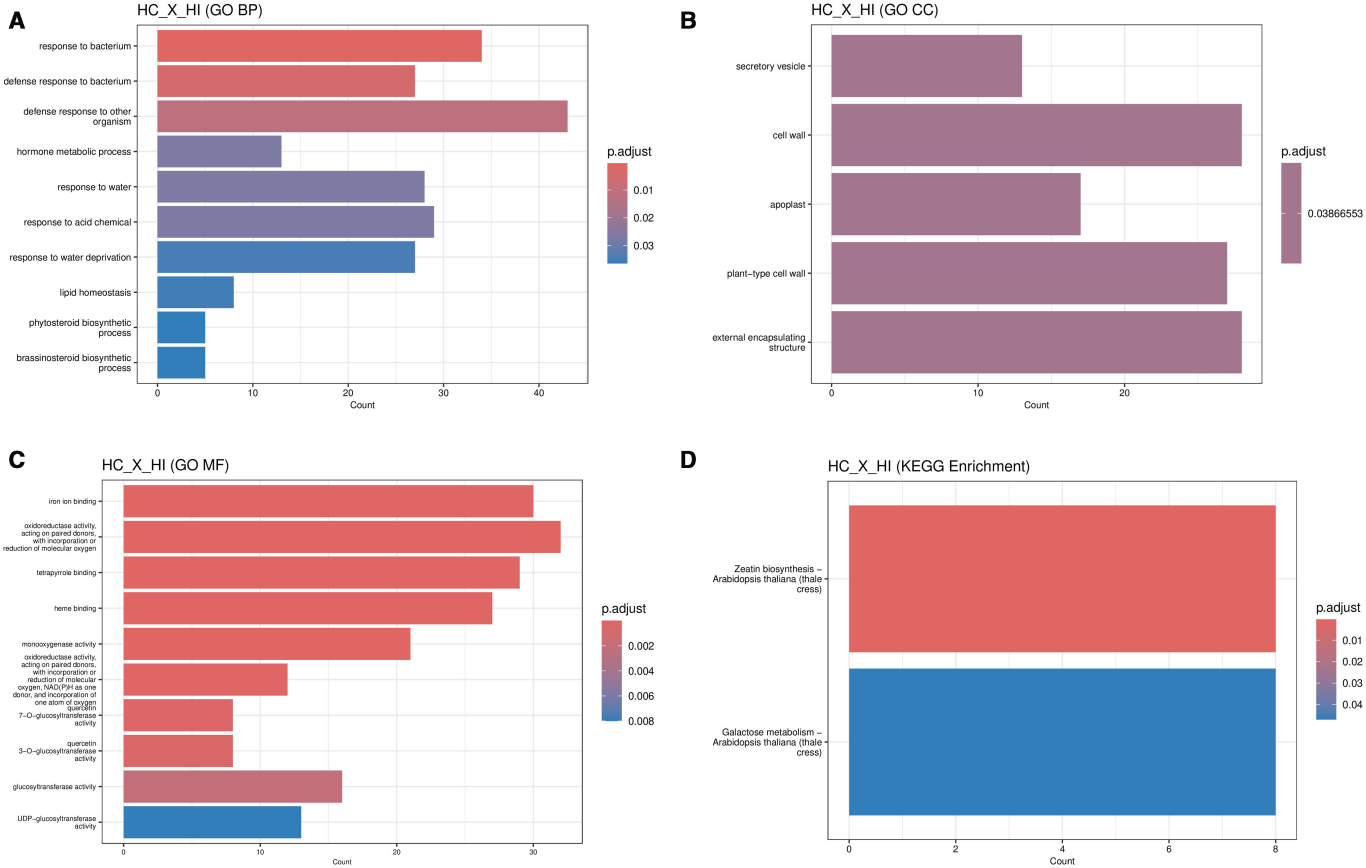
Gene Ontology (GO) enrichment analysis for differentially expressed genes between Sweet Harvest control (HC) vs. Sweet Harvest inoculated (HI) plants. Inoculation was done with *Pantoea ananatis* (PNA 97-1). **(A)** GO enrichment analysis for Biological Processes (BP) shows significant upregulation of defense-related processes, including response to bacteria and defense against other organisms, alongside processes related to water and hormone metabolism. **(B)** GO enrichment for Cellular Components (CC) highlights the involvement of genes associated with the cell wall, apoplast, and external encapsulating structures, suggesting structural reinforcement during infection. **(C)** GO enrichment for Molecular Functions (MF) indicates upregulation of genes involved in iron ion binding, oxidoreductase activity, and transferase activities, reflecting oxidative stress responses. **(D)** KEGG pathway enrichment analysis reveals significant activation of zeatin biosynthesis and galactose metabolism, pointing to hormonal regulation and cell wall remodeling efforts.

Comparison of susceptible Sweet Harvest control (HC) versus inoculated (HI) samples revealed 998 significantly differentially expressed genes, with 400 upregulated and 598 downregulated. Functional annotation of these genes showed widespread transcriptional activation of pathways involved in DNA binding, nucleotide metabolism, signal transduction, oxidative stress response, secondary metabolism, and multicellular development. GO enrichment indicated significant biological processes including “defense response to bacterium” “lipid homeostasis,” and “hormone metabolic process.” For instance, within the highly significant “defense response to bacterium” category (**Figure 4A**; **Supplementary Table S8**), the analysis identified the upregulation of key defense-related transcription factors such as ATWRKY54 and ATWRKY33, as well as the Cysteine-rich Receptor-like Kinase CRK8, which are all known components of plant immune signaling networks. The involvement of genes associated with the “cell wall”, “apoplast”, and “external encapsulating structure” (**Figure 4B**; **Supplementary Table S9**) suggests a transcriptional effort towards structural reinforcement. Key genes in this category include the xyloglucan endotransglucosylase XTH23, involved in cell wall remodeling, and the peroxidase PRX52, which can contribute to lignification and the oxidative burst, illustrating the molecular basis for this response. At the molecular level, the response was characterized by an enrichment of “oxidoreductase activity” and “iron ion binding” (**Figure 4C**; **Supplementary Table S10**), a response driven by the strong induction of numerous Cytochrome P450 family genes (e.g., CYP71B34, CYP81D8). Furthermore, we observed a specific enrichment for “UDP-glucosyltransferase activity”, exemplified by genes like UGT87A2 and UGT73B5, which are critical for modifying and detoxifying secondary metabolites during plant defense. KEGG pathway enrichment showed induction of “Galactose metabolism” and “Zeatin biosynthesis” (**Figure 4D**; **Supplementary Table S11**), suggesting hormonal and metabolic reprogramming during infection. This finding was further supported by the GO analysis, which identified an enrichment for ‘hormone metabolic process’ (**Figure 4A**), a category that included key zeatin biosynthesis genes such as the isopentenyltransferase ATIPT9 and the cytokinin-activating enzyme LOG7. Concurrently, the enrichment of ‘Galactose metabolism’ aligns with the GO analysis, pointing towards the mobilization of carbohydrates for cell wall polysaccharide synthesis.

**Figure 5:**
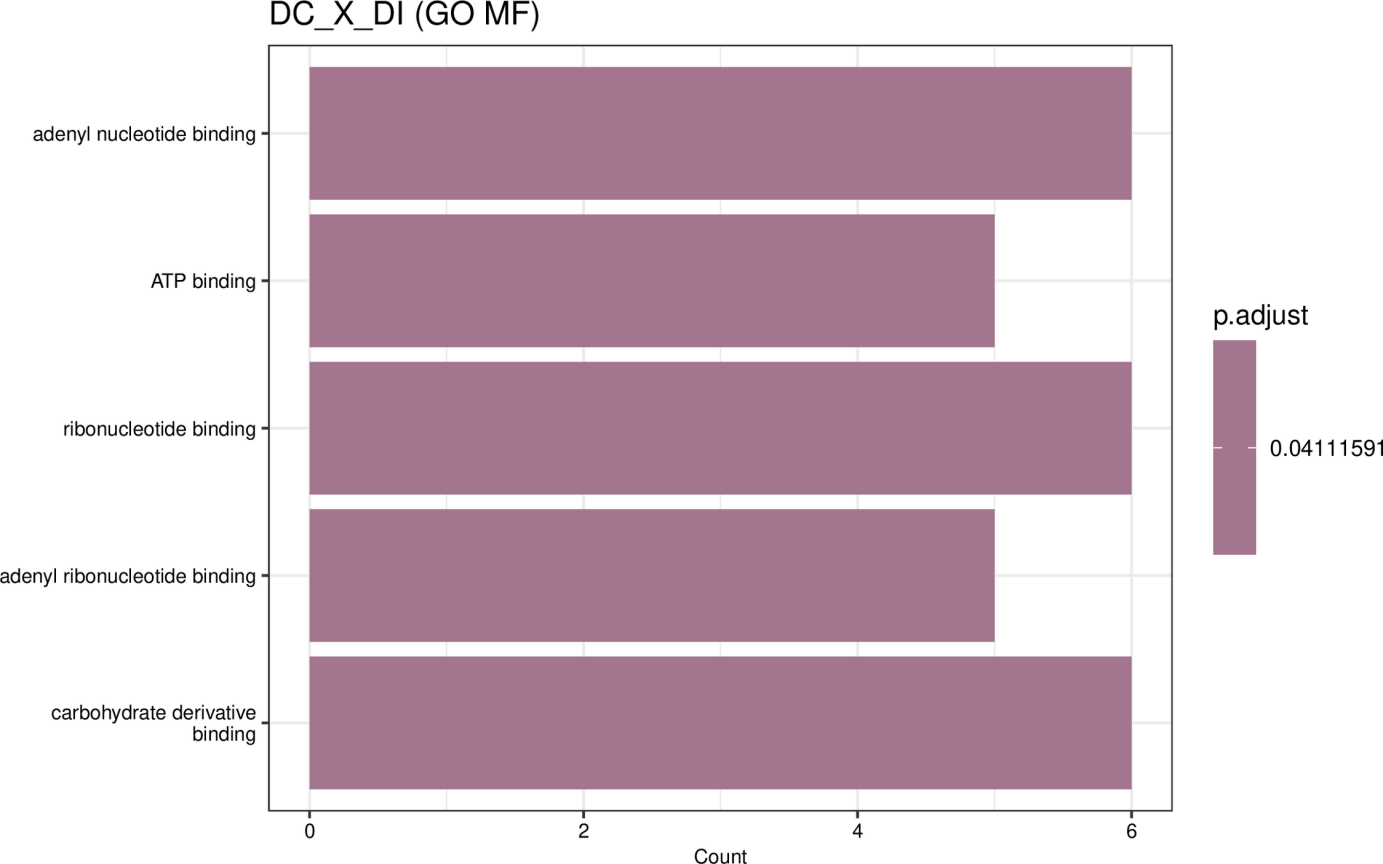
Gene Ontology (GO) enrichment analysis for differentially expressed genes between DPLD 19-39 control (DC) vs. DPLD 19-39 inoculated (DI) plants, with a focus on the molecular functions (MF) that are enriched in response to inoculation with *Pantoea ananatis* (PNA 97-1). GO enrichment analysis for MF reveals significant enrichment in genes associated with binding activities, including adenyl nucleotide binding, ATP binding, ribonucleotide binding, and carbohydrate derivative binding; these molecular functions are essential in energy transfer, nucleotide metabolism, and carbohydrate interactions, all of which are crucial during the plant’s response to pathogen stress.

**Figure 6:**
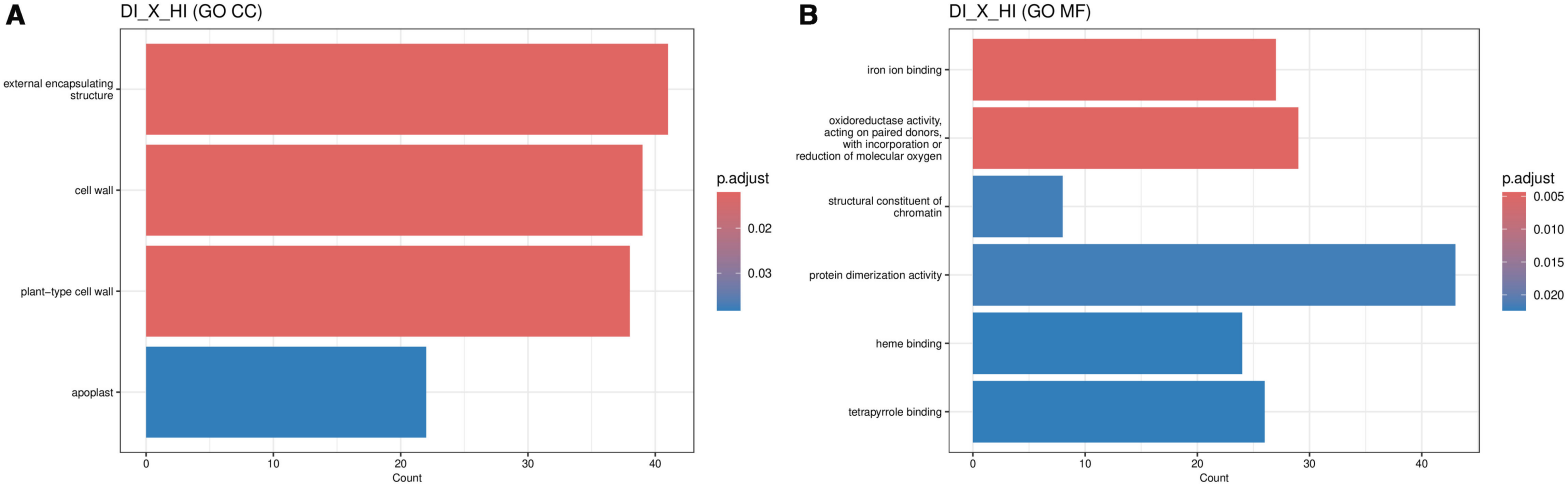
Gene Ontology (GO) enrichment analysis for differentially expressed genes between DPLD 19–39 inoculated (DI) vs. Sweet Harvest inoculated (HI) plants with *Pantoea ananatis* (PNA 97-1), highlighting cellular components and molecular functions that differ between the resistant and susceptible genotypes. **(A)** GO enrichment analysis for Cellular Components (CC) shows significant upregulation in genes associated with the external encapsulating structure, cell wall, plant-type cell wall, and apoplast in DPLD 19-39 compared to Sweet Harvest. This suggests that the resistant genotype, DPLD 19-39, enhances structural defenses during infection, potentially limiting pathogen spread by reinforcing its cell walls and apoplastic barriers. **(B)** GO enrichment analysis for Molecular Functions (MF) indicates enrichment in genes related to iron ion binding, oxidoreductase activity, and chromatin structural constituents, alongside protein dimerization and heme binding.

By contrast, the DPLD 19–39 genotype showed a more restricted transcriptional response, with only 57 significant differentially expressed genes identified. Of these, 30 were upregulated and 27 were downregulated in DI relative to DC (**Figure 4**). GO enrichment was limited to the molecular function category, highlighting a focused response related to metabolic activation. The analysis revealed significant enrichment in terms such as “ATP binding”, “adenyl nucleotide binding”, and “carbohydrate derivative binding” (**Figure 5**; **Supplementary Table S12**). This enrichment was driven by the upregulation of key genes involved in energy metabolism and cellular maintenance under stress, such as the ATP-dependent protease LON1 and isovaleryl-CoA dehydrogenase ATIVD. The induction of G6PD1 (glucose-6-phosphate dehydrogenase) is particularly noteworthy, as it is a rate-limiting enzyme of the pentose phosphate pathway, essential for generating the reducing power (NADPH) required for antioxidant defense. These findings suggest that the resistant genotype rapidly mobilizes metabolic resources to fuel a robust and efficient defense response upon pathogen recognition. This focused metabolic activation, which did not result in the significant enrichment of any broad KEGG pathways, prompted a direct comparison with the susceptible genotype to understand their differing induced defense programs. Therefore, an analysis between inoculated resistant (DI) and inoculated susceptible (HI) plants identified 1577 differentially expressed genes, with 698 upregulated in DI and 879 in HI (**Figure 6**). GO enrichment indicated major functional divergences between the two genotypes’ responses. In the resistant DPLD 19-39, there was a significant upregulation of genes associated with cellular components like the “external encapsulating structure” and “plant-type cell wall” (**Figure 6A**; **Supplementary Table S13**). This response was driven by genes essential for structural defense, including the xyloglucan endotransglucosylase AtXTH22 and the pectin methylesterase PME53, underscoring a strategy of rapid cell wall reinforcement. At the level of molecular function (**Figure 6B**; **Supplementary Table S14**), the DPLD 19–39 response was characterized by a strong enrichment in “iron ion binding” and “oxidoreductase activity”, exemplified by the induction of Cytochrome P450s such as CYP96A1 and CYP81D2. Interestingly, a unique enrichment for “structural constituent of chromatin”, including several core histone genes (e.g., H2B, H3.3), was also observed. This suggests that the resistant genotype undergoes significant transcriptional reprogramming, potentially involving chromatin remodeling, to orchestrate its distinct and effective defense program, which is fundamentally different from the response mounted by the susceptible genotype.

**Figure 7:**
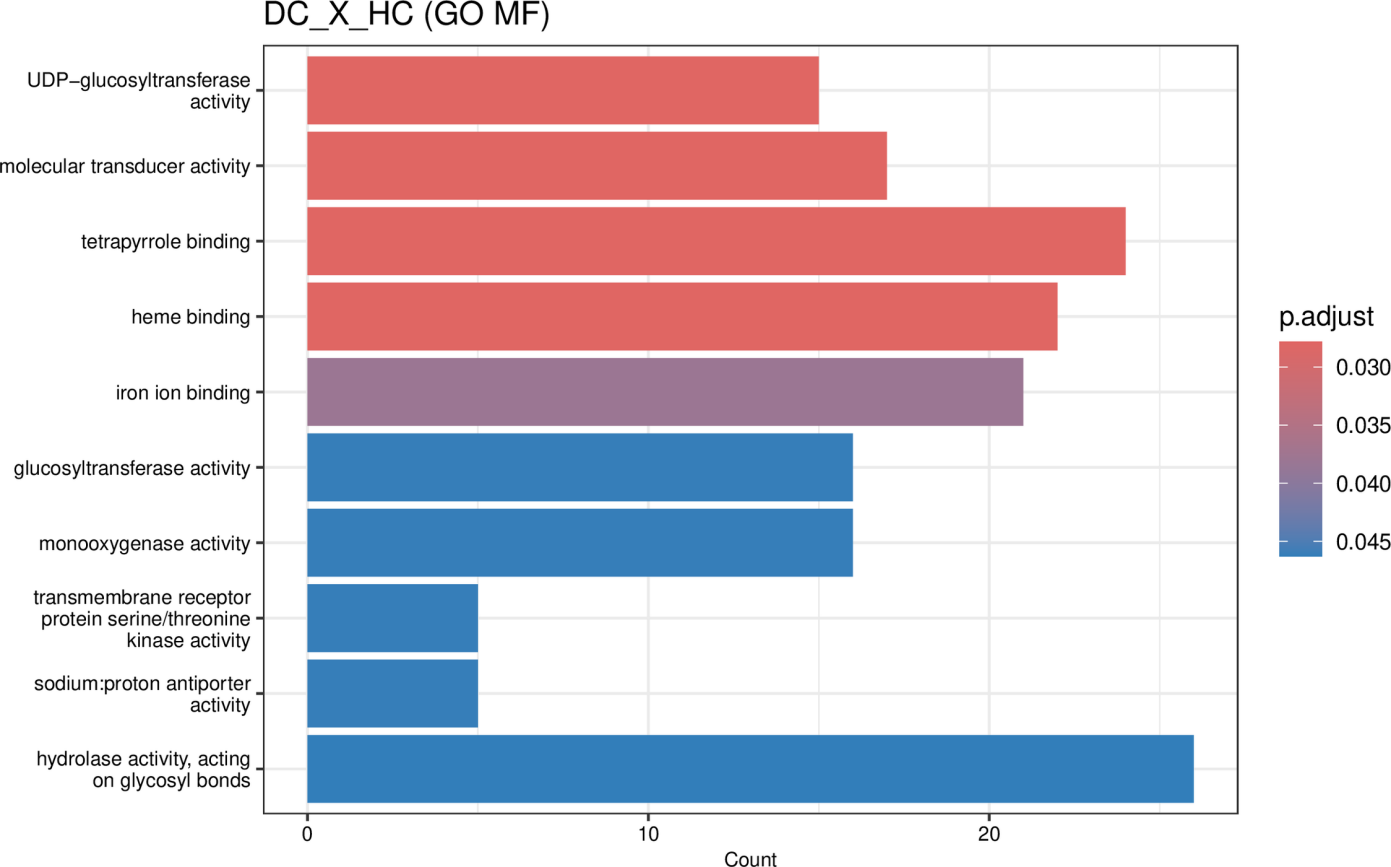
Gene Ontology (GO) enrichment analysis for differentially expressed genes between DPLD 19–39 control (DC) vs. Sweet Harvest control (HC) plants, highlighting molecular functions enriched in the resistant and susceptible genotypes under non-infected conditions. GO enrichment analysis for Molecular Functions (MF) shows significant upregulation in DPLD 19-39 of genes related to UDP-glucosyltransferase activity, molecular transducer activity, and iron ion binding.

Even in the absence of infection, 1254 genes were differentially expressed between DPLD 19–39 and Sweet Harvest, indicating that basal, pre-formed characteristics contribute to resistance. A GO enrichment analysis of these genes (**Figure 7**; **Supplementary Table S15**) revealed that the resistant DPLD 19–39 genotype shows constitutive upregulation of genes related to key defense-ready functions. Notably, there was a strong enrichment for “UDP-glucosyltransferase activity”, driven by multiple UGT family genes such as UGT74D1 and UGT88A1, which are critical for conjugating and storing defensive secondary metabolites. Additionally, we observed enrichment for “molecular transducer activity”, including several receptor-like kinases (e.g., HERK1, AtCERK1) and signaling components, suggesting a heightened state of environmental perception. Finally, terms like “iron ion binding” were also enriched, pointing to constitutive differences in the expression of Cytochrome P450s (e.g., CYP90D1, AtDWF4) and peroxidases involved in metabolic and redox homeostasis. Collectively, these basal differences suggest that DPLD 19–39 exists in a state of enhanced biochemical and signaling preparedness, allowing for a faster and more effective response upon pathogen challenge. Relative to Sweet Harvest under infection, the resistant background shows higher cellulose synthase expression and reduced wall proteolysis, together with a ROS burst–with–quenching signature and fast perception/damage control. In DI vs HI, g499569 and g223739 (cellulose synthase) are significantly increased (p-value<0.05), whereas the wall-loosening subtilase g358329 is significantly decreased (p-value<0.05), coherent with active wall tightening (Cheng et al., 2021; Bethke et al., 2014; Ma, 2024) and potentially reduced apoplastic porosity (Cheng et al., 2021; Bethke et al., 2014). Apoplastic ROS formation and enzymatic quenching are supported by significant induction (p-value<0.05) of peroxidase g497991, ascorbate peroxidase g488970, and catalase g283552, consistent with a high-amplitude, short-duration ROS pulse followed by rapid termination (Lee et al., 2020b; Torres et al., 2006; Almagro et al., 2009). Perception and controlled resolution are reflected by LRR-RLK-like g316766, LRR-RLK-like g424831, and decreased ATG18h g473595, aligning with wall/apoplast and oxidoreductase enrichments while enabling rapid cleanup of damaged compartments (Kang et al., 2022).

In DC vs DI, nodes that tune hormone and apoplastic physiology are prominent, with JAZ1-like g319478 showing significant increased expression (p-value<0.05) while OPR4 g364481 is decreased; UGT g141132 and TIP2 aquaporin g290555 are also significantly increased, consistent with a pulse-and-containment logic rather than sustained hormone outputs (Torres et al., 2006; Wei et al., 2022).

**Figure 8:**
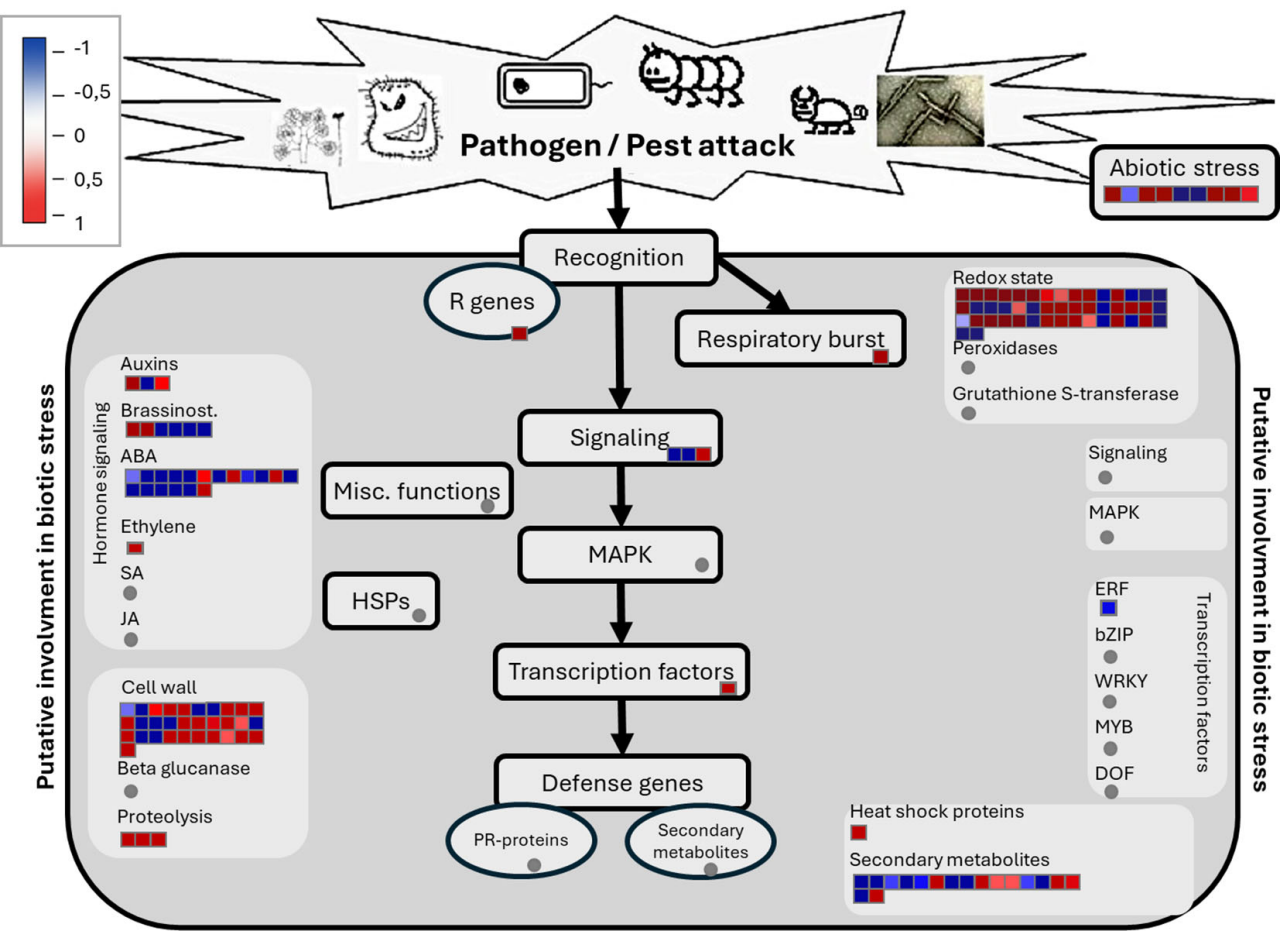
Main biotic stress pathways identified in resistant *Allium cepa* using MapMan software (Thimm et al., 2004). This molecular pathway analysis compares differentially expressed genes with statistical significance (DEASS) between DPLD 19-39 (resistant) and Sweet Harvest (susceptible) in response to *Pantoea ananatis* (PNA 97-1) infection. Red boxes indicate up-regulated genes, while blue boxes indicate down-regulated genes. Light gray boxes indicate specific molecular pathways, with the total genes belonging to that specific pathway.

To visualize these findings at a systems level, we used MapMan to organize differentially expressed genes by functional category (**Figure 8**). This analysis provides a clear illustration of the defense program in DPLD 19-39. A central component of this response is the activation of the ‘Redox state’ pathway, where the sub-bin for ‘Peroxidases’ shows a majority of its constituent genes are upregulated (indicated by the prevalence of red squares). This represents the strong induction of essential ROS-scavenging enzymes like PER64 and PRX52. The “Cell wall” pathway is also modulated, showing numerous up- and downregulated genes (a mix of red and blue squares), consistent with active reinforcement and remodeling, a process involving genes like the xyloglucan endotransglucosylase AtXTH22.

Critically, the MapMan visualization highlights a distinct hormonal signaling signature. The pathways for classical defense hormones, Salicylic Acid (SA) and Jasmonic Acid (JA), show no significant changes (gray circles). In contrast, the ‘Ethylene’ pathway is activated (red square), indicating its importance in this interaction. This specific hormonal profile corresponds to a complex downstream modulation of “Transcription factors”. While specific members of the ERF and bZIP families are shown as downregulated in this visualization, the overall activation of the ethylene pathway suggests a broader reprogramming of gene expression. This integrated view underscores a defense strategy that prioritizes structural barriers and proactive redox stress management, orchestrated by non-classical hormonal signaling pathways.

**Figure 9:**
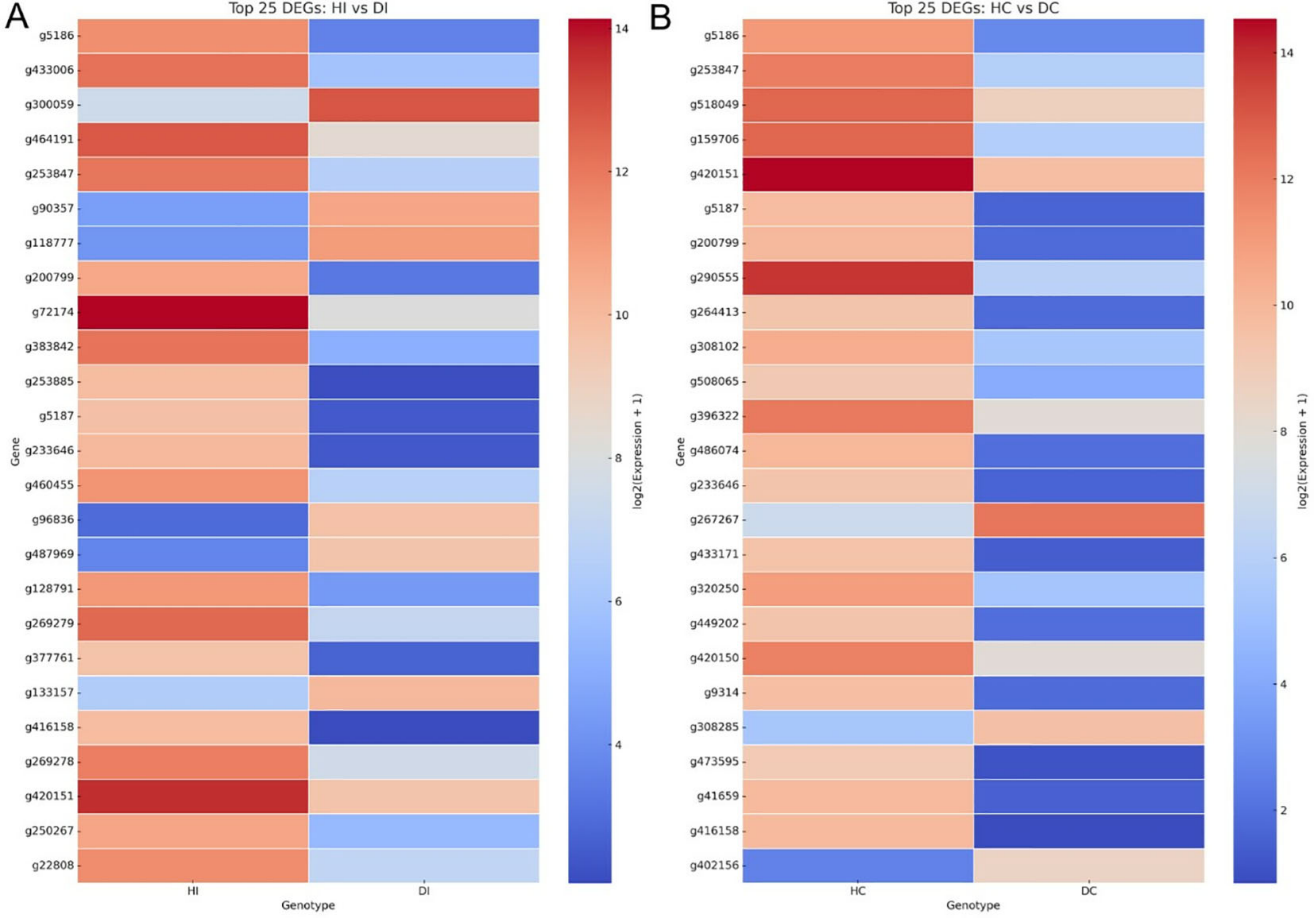
Heatmap visualization of the top 25 differentially expressed genes (DEGs) from transcriptome comparisons of Sweet Harvest and DPLD 19-39. **(A)** DEGs identified between PA-inoculated Sweet Harvest (HI) and PA-inoculated DPLD 19-39 (DI), revealing distinct expression patterns in the resistant genotype. **(B)** DEGs identified between control Sweet Harvest (HC) and control DPLD 19-39 (DC), reflecting constitutive differences in gene expression. Colors indicate scaled expression (log_2_ fold-change + 1), with red indicating upregulation and blue indicating downregulation.

Together, this systems-level view provided by the MapMan analysis (**Figure 8**) and the gene-specific expression patterns visualized in the heatmap (**Figure 9**; **Supplementary Table S6**) provide a comprehensive summary of the distinct defense strategies deployed by each genotype.

**Figure 10:**
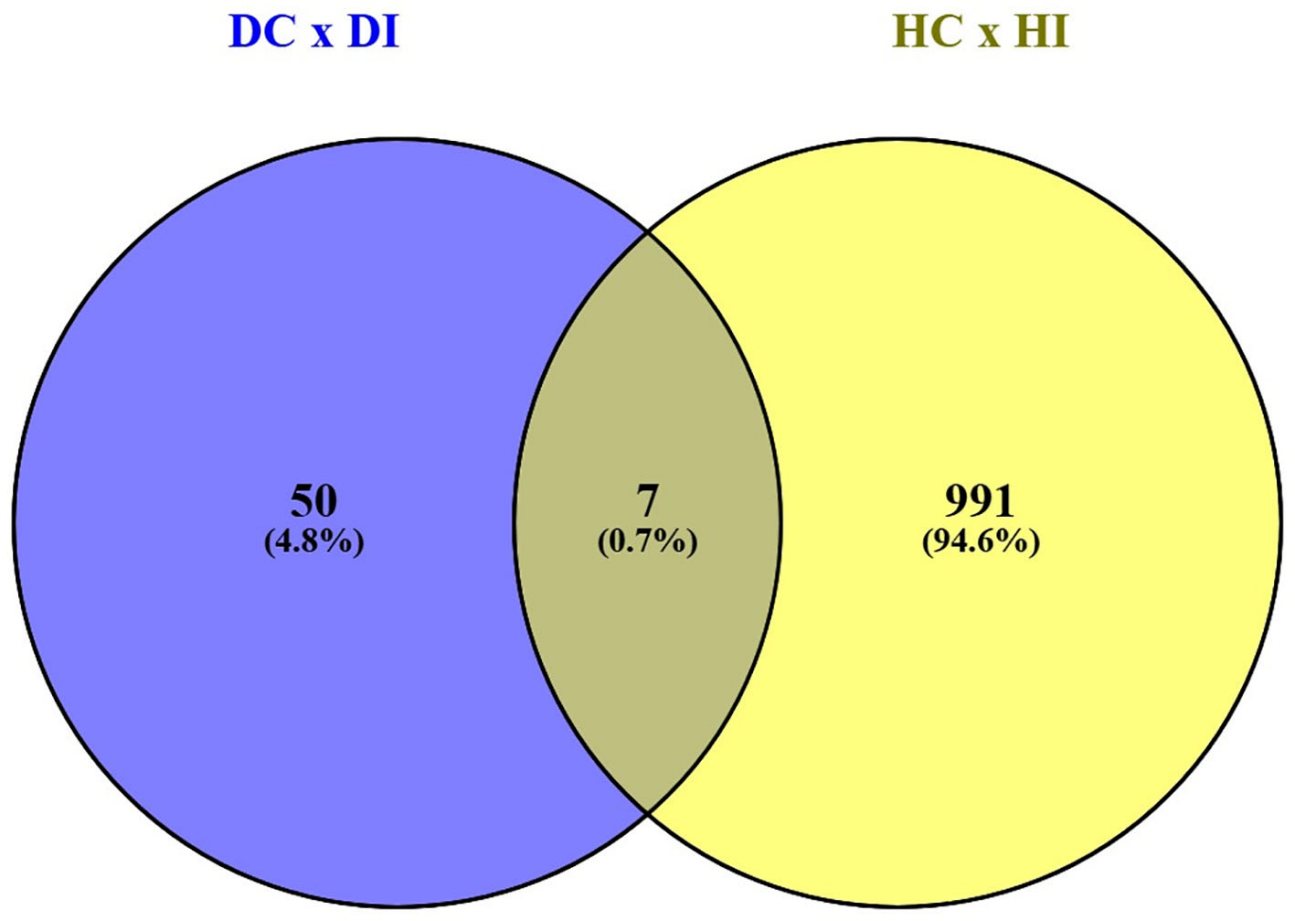
Venn diagram showing the overlap between differentially expressed genes (DEG, p-value<0.05) found in both DPLD 19-39 control (DC) vs. DPLD 19-39 inoculated (DI) plants and between Sweet Harvest control (HC) vs. Sweet Harvest inoculated (HI) plants.

The divergence between these defense strategies is quantitatively illustrated by a comparison between the sets of genes induced in each genotype (DC vs. DI and HC vs. HI). This analysis was found that only 7 differentially expressed genes were common between these sets. In comparison, 991 differentially expressed genes were unique to HC × HI and 50 differentially expressed genes were unique to DC × DI (**Figure 10**). The list of these genes are listed in the **Supplementary Table S7**.

## Discussion

Our extensive phenotypic assessment of 982 *Allium* genotypes, including various species from the Allium genus *A. cepa*, *A. cepa* var. *cepa*, and *A. fistulosum*, indicated substantial phenotypic differences in resistance to PA strain PNA 97-1. Both field and greenhouse evaluations revealed that specific genotypes consistently displayed lower disease severity, as measured by the AULPC. Notably, the *A. fistulosum* genotype “Zhang Qiu Da Cong” and the *A. cepa* genotype “DPLD 19-39” demonstrated moderate to high levels of resistance, with significantly lower AULPC values than the highly susceptible cultivar Sweet Harvest. These findings are consistent with previous reports of variable resistance to other pathogens (e.g., *Fusarium oxysporum*) across *Allium* species and wild relatives (*A. roylei*, *A. fistulosum*) (Khrustaleva and Kik, 2000; Havey et al., 2004). Although *A. fistulosum* contained the largest number of resistant genotypes (*n* = 18), this remains a surprisingly low proportion of the total panel screened.

Greenhouse and growth chamber experiments reinforced these results, demonstrating that PA did not cause systemic infection in DPLD 19–39 but did so in Sweet Harvest. The consistent display of resistant phenotype in DPLD 19-39, including reduced bulb colonization, supports its utility in breeding programs aimed at enhancing resistance against pantaphos producing PA. Thus in order to make deliverable impact through breeding programs, several steps are essential. First, validate resistance across diverse environment and diverse pantaphos-containing PA strains. Thrips-mediated transmission of these bacterial strains under field conditions should also be considered (Dutta et al., 2014b; Polidore et al., 2021). Next, conduct crossing and pre-breeding by making crosses between DPLD 19–39 and elite lines (short/intermediate-day) to introgress resistant traits while preserving agronomic traits of the elite lines (Khrustaleva and Kik, 2000).

Transcriptomic analysis of the resistant (DPLD 19-39) and susceptible (Sweet Harvest) genotypes provided mechanistic insights into resistance. In DPLD 19-39, genes related to cell wall structural molecule activity and nucleotide binding were upregulated, suggesting that resistant plants reinforce their cell walls in response to infection, likely as a countermeasure to pathogen-derived cell wall–degrading enzymes (CWDEs) such as pectate lyases, although these enzymes are absent in PA strain 97-1 (Flors et al., 2008; Ponce de León and Montesano, 2013; Wang et al., 2021). Additionally, control and infected resistant plants exhibited elevated gene expression in redox regulation, including catalases and peroxidases, suggesting that resistant plants not only produce reactive oxygen species (ROS) to signal defense but also possess the enzymatic machinery to manage oxidative damage (Torres et al., 2006).

Hormonal signaling also emerged as a significant component of resistance. Genes linked to ethylene (ET), abscisic acid (ABA), auxin, and brassinosteroids were significantly enriched in DPLD 19-39, suggesting alternative defense signaling pathways outside of classical salicylic acid (SA) and jasmonic acid (JA) routes. These hormones potentially reinforce the cell wall, regulate programmed cell death (PCD), and promote stomatal closure to restrict bacterial ingress (Broekgaarden et al., 2015).

While classical SA/JA outputs were not prominent in this system, the ET/ABA/auxin/BR signature in DPLD 19–39 is consistent with modulation of the two defense axes: cell wall tightening and pulse-and-quench ROS control. In DI vs HI, cellulose synthase A (CESA) genes (g499569, g223739) increase while the wall-loosening subtilase g358329 decreases, and redox enzymes (POX g497991, APX g488970, CAT g283552) support a brief, localized ROS burst followed by enzymatic quenching; the hormone-linked nodes that shift with genotype (JAZ1-like g319478↑, OPR4 g364481↓, UGT g141132↑, TIP2 aquaporin g290555↑, with LRR-RLKs g316766/g424831) are congruent with that containment-oriented program. Thus, we interpret the ET/ABA/auxin/BR changes as plausible modulators of wall reinforcement and ROS homeostasis in DPLD 19-39, while noting that the specific hormone–gene couplings in *A. cepa*-pantaphos remain to be resolved.

Furthermore, our three orthogonal contrasts: susceptible HC vs HI, resistant DC vs DI, and the direct infected comparison DI vs HI coherently support a containment-oriented defense circuit in DPLD 19–39 that explains confined lesions without over-reliance on diffuse SA/JA outputs.

At the gene level, the resistant response in DPLD 19–39 is marked by coordinated wall reinforcement and redox control alongside targeted regulatory tuning. In DI vs HI, cellulose synthases g499569 and g223739 are significantly elevated while the wall-loosening subtilase g358329 is reduced, consistent with rapid wall tightening and decreased apoplastic permeability. In parallel, a pulse-and-quench redox program is evident from the induction of peroxidase g497991, ascorbate peroxidase g488970, and catalase g283552, supporting localized ROS cross-linking followed by enzymatic cleanup. Perception and regulatory control are reflected by LRR-RLK-like g316766 and g424831, and by genotype-specific tuning of hormone-linked nodes (JAZ1-like g319478 increased; OPR4 g364481 decreased), together with UGT g141132 and TIP2 aquaporin g290555, a configuration consistent with containment rather than diffuse SA/JA amplification. The DI vs HI enrichment for “structural constituent of chromatin” (e.g., H2B, H3.3) further suggests chromatin-level facilitation of this program. Collectively, these candidates provide a concrete mechanism: wall stiffening plus localized, rapidly quenched ROS under fast perception/regulatory control, which explains confined lesions in DPLD 19–39 and yields testable targets for breeding and follow-up validation (with qPCR trends consistent in direction, where assessed).

In DI vs HI, DPLD 19–39 significantly elevates cellulose synthases (g499569, g223739) and depresses wall proteolysis (g358329, subtilase), consistent with rapid wall tightening and reduced apoplastic permeability (Cheng et al., 2021; Bethke et al., 2014; Ma, 2024). In the susceptible background under infection (HC vs HI), PME-like g87538 and PR-like g137806 are reduced, while metabolism diverts toward sterols (g269279, cycloartenol synthase) and sulfate transport (g540144), a pattern not aligned with physical containment (Cheng et al., 2021; Bethke et al., 2014; Ma, 2024).

The comparison between Di vs HI shows significant induction of peroxidase g497991, ascorbate peroxidase g488970, and catalase g283552, supporting a high-amplitude, short-duration apoplastic ROS pulse for signaling and polymer cross-linking, followed by enzymatic quenching to limit collateral damage (Lee et al., 2020a; Torres et al., 2006; Almagro et al., 2009).

The resistant line’s genotype-specific tuning (from DC vs DI) reinforces this hypothesis that JAZ1-like g319478 rises the expression while OPR4 g364481 reduces the expression, with both UGT g141132 and TIP2 g290555 are elevated. These features naturally couple to NADPH-dependent cycles that both generate and detoxify ROS (Torres et al., 2006; Wei et al., 2022).

Taken together, DPLD 19–39 engages wall stiffening, localized ROS pulses plus quenching, and NADPH-supported enzymatic buffering with fast perception and damage resolution (Kang et al., 2022), offering a mechanistic narrative for lesion containment that is not captured by hormone titers alone.

What distinguishes DPLD 19–39 is not simply the presence of wall reinforcement or ROS activity, processes observed across many plant–pathogen systems, but the specific way these responses are deployed against pantaphos-producing PA. Unlike the diffuse SA/JA amplification typical of necrotroph defense, DPLD 19–39 mobilizes a fast, localized wall-tightening and ROS pulse-with-quench program, coupled with chromatin-level facilitation and hormone tuning through ET/ABA/auxin/BR. This containment-oriented strategy restricts necrosis to small, bounded lesions and prevents systemic bulb rot, a pattern not reported in prior *Allium* transcriptomic studies. Together, these features highlight that resistance in DPLD 19–39 is not generic but reflects a distinct, pantaphos-specific adaptation that offers testable breeding targets.

Because our analysis is gene-level rather than isoform-resolved, we interpret these effects as coordinated regulation of gene families that implement wall tightening and ROS pulse-and-quench dynamics, which is sufficient to explain the resistant phenotype without invoking isoform-specific investigations that could also complement our results.

Notably, genes involved in extracellular matrix remodeling and PCD underwent upregulation, implying that DPLD 19–39 restricts pathogen spread via spatial containment of necrotic tissue. Secondary metabolism also appeared to play a role, with enrichment in genes linked to phenolic biosynthesis and other defensive compounds. There was no evidence for any upregulation of C-P lyase genes, which are typically responsible for degrading phosphonates, suggesting that resistance is not due to pantaphos degradation. Instead, the plant may rely on physical barriers and oxidative containment to mitigate the effects of the toxin (Coutinho and Venter, 2009; Polidore et al., 2021).

Beyond generic categories, our contribution is a specific configuration in *A. cepa* under *in vivo* challenge by pantaphos-producing PA. We showed that reduced lesion development or progression is paired with a transcriptome program that tightens the cell wall (increase of CESA/peroxidases, decrease of subtilases) and executes a pulse-and-quench ROS dynamic (APX/CAT/POX) without SA/JA predominance (Torres et al., 2006; Almagro et al., 2009; Bethke et al., 2014). This aligns with the phosphonate-driven necrosis of center rot (Asselin et al., 2018; Polidore et al., 2021) and sets testable predictions, including that purified pantaphos should suffice to trigger tightening and quenched ROS, that *in planta* wall mechanics/porosity should shift in resistant vs. susceptible genotypes, and that perturbing chromatin-linked regulators should relax the tightening/ROS program.

Future research should further characterize the genetic basis of resistance to pantaphos PA, including the possible discovery of novel genes conferring insensitivity to pantaphos. Continued transcriptomic and functional validation in resistant genotypes will support durable resistance breeding and long-term onion crop sustainability (Khandagale et al., 2022; Prajapati et al., 2023).

## Conclusions

In this study, we identified a resistant *A. cepa* genotype, DPLD 19-39, that consistently exhibited reduced symptoms of PA (PNA 97-1) infection in both foliar and bulb assays under field, greenhouse, and growth chamber conditions. Comparative transcriptome analysis between DPLD 19–39 and the susceptible genotype Sweet Harvest revealed that resistance may be associated with enhanced cell wall fortification, regulation of oxidative stress, and activation of defense-related pathways. These findings provide a foundation for future efforts to characterize genetic resistance to PA and offer valuable targets for breeding more resilient onion cultivars.

## Data Availability

The transcriptome sequencing data is available at NCBI, BioProjectaccession code PRJNA1293386.
